# Hortus Medicus, or Figures and Descriptions of the More Important Plants Used in Medicine, or Possessed of Poisonous Qualities; with Their Medical Properties, Chemical Analysis, &c. &c.

**Published:** 1834-07-01

**Authors:** 


*Hortus Medicus; or, Figures and Descriptions of the more important Plants used in Medicine, or possessed of Poisonous Qualities; with their Medical Properties, Chemical Analysis, &c. &c.* By GEORGE GRAVES, F.L.S. &c., and JOHN DAVIS MORRIS, M.D. &c.—Edinburgh and London, 1834. 4to. pp. 274; thirty-eight coloured Plates.

THE figures of this work are beautiful, both in drawing and colouring; and the volume, on the whole, has a very prepossessing appearance. Hence we anticipated, on commencing its perusal, that our task would be one of unmixed praise. But, alas! human works, like human lives, are webs of mingled yarn; and, notwithstanding our prepossessions in its favour, we have been disappointed with the Hortus Medicus, because it has failed to realize those expectations which its titlepage and preface allowed, nay, led us to indulge.

The Hortus Medicus is declared to contain "*figures* and *descriptions* of the more important plants used in medicine or possessed of poisonous qualities;" and the preface informs us that "the expensive forms and extent of the modern works on Medical Botany induced the authors to attempt the present publication, in which such plants only are introduced as are in present use by British practitioners, or, from being endued with poisonous qualities, are likely to call for the attention of medical men, to remedy or alleviate their effects."

This all sounds very fairly, and the scheme is plausible. Looking at the voluminous medical Floras of Germany, and even those of our own country, (perhaps in some respects too prolix,) we should commend the publisher who would select "the more important plants used in medicine" from those of less value, or which have ceased to be employed, and give in their stead figures and descriptions of such as are "possessed of poisonous qualities." But then we should expect to have figures of *all* the more important medical plants, especially of those that are rare and *most* important; and the chief of the poisonous species, particularly the indigenous ones, should be also given. The want of this offends us in the Hortus Medicus; for, although between 180 and 200 species are mentioned, which, from their being selected and described, we presume have been considered as the more important and poisonous species, yet of these less than fifty are figured; so that the volume, instead of being a "*Hortus Medicus*," in the proper sense of the words, is rather "a compilation on materia medica, with figures of a few of the plants employed in medicine, or known as poisons."

In the next place, we come to consider the principles of the selection; and, when so few plants are figured, we of course expect they should be the *more*, if not the *most* important. Here, again, we are disappointed; for certainly *Papaver somniferum* is a more important officinal plant than *Papaver Rhœas*, and yet the latter is figured, and the former not. Again, why should the burdock *(Arctium Lappa)*, and the buck-bean *(Menyanthes trifoliata)*, and others of equal non-importance, have each of them a whole plate dedicated to their representation, while the *Laurels* (cassia, cinnamon, and camphor,) the *Peppers*, the *Tobacco*, the *Jalap*, the *Scammony*, the *Saffron*, *Ginger*, *Cardamom*, *Ipecacuan*, *Capsicum*, *Calomba*, *Cascarilla*, *Aloe*, *Rhubarb*, *Squill*,

*Gamboge, Gentian, Hop, Nutmeg, Clove, Allspice, Ricinus, Smilax, Croton Tiglium, and Eleutheria,* &c. are omitted. We have every proper respect for the *Dandelion,* but we had rather have had figures of the different species of *Cinchona,* showing their leaves and flowers. We do not mean to detract aught from the importance of *Linum catharticum,* but we think it might well have given place to the *Senna.* Doubtless the *Bistort* and the *Mezereon* should have been figured; but then the whole of the *Pines* and the *Acacias* should not have been left out. It may have been very right to give representations of the common mallow *(Malva sylvestris),* and the black and white *Mustards,* with the broom *(Cytisus scoparius),* and two species of *Sium,* or water parsnep; but surely they are not more important plants as medicines, nor more fatal ones as poisons, than the white hellebore *(Veratrum album),* the hedge hyssop *(Gratiola officinalis),* the wolf-bane *(Arnica montana),* the cajeput *(Melaleuca Cajeputi),* the serpentary *(Serpentaria Virginica),* the willow *(Salix Russelliana* or *Capræa),* the poison ash *(Rhus Toxicodendron),* the nux vomica *(Strychnos),* and others too numerous to catalogue.

One reason alone suggests itself to us for this arbitrary selection, in which we should say many of the *more* important plants are left unfigured, viz. that those were sketched which came readiest to hand, and which, of course, from being the most familiar and best known, would the least be wanted for the instruction of the student: for, what other cause can account for a figure being given of the *Bryony,* while both the *Elaterium* and the *Colocynth* are omitted?

We say no more upon the head of omissions; but, unpleasant as is the task of finding fault with a work in some parts excellent, still it is a duty we owe to the public not to let vessels sail on the literary seas under false colours; and if, in an appendix, which might form a second volume, the author will supply the defects of the present one, we shall then cordially recommend his work to the student; although, if rendered as copious in illustrations as the other medical Floras to which he alludes, we doubt whether it would be much inferior to them in cost.

In fine, notwithstanding the above named serious omissions the volume is valuable, for the plates already given are beautiful, both as representations of nature and works of art; and the medical and chemical notes are creditable to the compiler.

